# The role of the S1 gene in glandular stomach pathogenesis and tropism of infectious bronchitis virus H120 strain in poultry

**DOI:** 10.1128/spectrum.00008-25

**Published:** 2025-08-12

**Authors:** Zhenkai Dai, Yutao Teng, Jiabei Song, Zhengzhong Xiao, Jing Zhang, Keyu Feng, Guanming Shao, Xinheng Zhang, Qingmei Xie

**Affiliations:** 1 State Key Laboratory of Swine and Poultry Breeding Industry & Guangdong Laboratory for Lingnan Modern Agriculture, College of Animal Science, South China Agricultural University 12526, Guangzhou, P. R. China; 2 School of Biology and Agriculture, Shaoguan University 47888, Shaoguan, P. R. China; 3 Guangdong Provincial Key Lab of AgroAnimal Genomics and Molecular Breeding, College of Animal Science, South China Agricultural University 12526, Guangzhou, P. R. China; 4 Guangdong Engineering Research Center for Vector Vaccine of Animal Virus, Guangzhou, P. R. China; 5 Zhongshan Innovation Center of South China Agricultural University, Zhongshan, P. R. China; Barnard College, Columbia University, New York, New York, USA

**Keywords:** Infectious bronchitis virus, H120 strain, S1 gene, glandular and muscular stomach

## Abstract

Infectious bronchitis virus (IBV) H120 strain, a widely used vaccine in poultry production, has been associated with an increased incidence of avian glandular and muscular stomach inflammation. This study aimed to investigate the pathogenic factors of stomach inflammation induced by the IBV H120 vaccine and identify genetic regions affecting glandular and muscular stomach lesions through the construction of recombinant strains. Our findings revealed that the H120 strain exhibits a more pronounced tropism for the glandular and muscular stomach compared to the 4/91 strain. Notably, the replacement of the S1 gene in the H120 strain resulted in reduced pathogenicity in the chicken’s glandular stomach. These results suggest that the S1 gene of the H120 strain plays a critical role in viral replication within the chicken stomach, contributing to the development of muscular and glandular stomach lesions. This research provides valuable insights into the pathogenesis of IBV and offers potential strategies for optimizing vaccine strains by targeting the S1 gene to minimize viral load in the glandular and muscular stomachs, ultimately improving poultry health and productivity.

**IMPORTANCE:**

Infectious bronchitis virus (IBV) causes high morbidity and mortality worldwide, leading to significant economic losses. While the widespread vaccination with the H120 strain has effectively curbed the spread of IBV, we have discovered that the H120 strain can cause glandular stomach inflammation in chickens. Further research indicates that the S1 gene of the H120 strain determines its tropism for the glandular stomach. Studying the glandular tropism of the H120 strain is crucial for developing effective and safe IBV vaccines.

## INTRODUCTION

Infectious bronchitis is an acute, highly transmissible respiratory disease induced by the infectious bronchitis virus (IBV), with the potential to extensively spread across the globe, profoundly affecting global poultry industry growth (1). IBV exhibits extensive diversity in both serotypes and genotypes. This variation, coupled with the broadening transmission scope, viral mutational progression, and artificial immunization practices, has spurred an increase in the genetic mutation rate of IBV. Initially, IBV was recognized solely as a cause of respiratory ailments, manifesting signs such as coughing, nasal discharge, and tracheal sounds (2, 3). Nevertheless, research has since revealed a more comprehensive tissue affinity for IBV, with clinical phenotypes encompassing renal, intestinal, glandular stomach, and reproductive system forms. The H120 strain of IBV serves as a prevalent vaccine in poultry farming, offering robust protection against a range of IBV serotypes. Yet, recent findings have highlighted a rise in conditions such as inflammation of the glandular stomach among flocks vaccinated with H120, prompting worries about its potential pathogenic implications (4). IBV pathogenesis is multifaceted, involving intricate interplay between viral components and host factors that collectively influence tissue tropism, viral replication, and immune system evasion (5, 6). Central to IBV infection is the viral spike (S) glycoprotein, composed of S1 and S2 subunits. This glycoprotein plays a pivotal role in facilitating receptor binding, promoting membrane fusion, and determining viral serotype specificity (7).

The S protein is one of the most crucial structural proteins of the IBV and is also the largest, with a molecular weight of approximately 150–200 kDa. It performs multiple functions, including playing a decisive role in the virus’s entry into host cells, interaction with host cells, and immune evasion during various processes (8, 9). Typically, the S protein is cleaved into two subunits: the N-terminal S1 and the C-terminal S2, after being recognized by a basic cleavage site (10). As the main epitope of the coronavirus, the S protein carries receptor-binding sites. When IBV infects host cells, the S1 protein binds to the host cell receptor, mediating virus attachment and reducing the binding capacity between S1 and S2. This separation of the S1 subunit from the S2 subunit induces the fusion of the viral envelope with the host cell membrane, releasing the IBV genome into the host cell (11). Therefore, the pathogenicity and infectivity of IBV are determined by the S1 protein (12). Within the entire viral genome, the S1 gene has the highest mutation rate, and the genetic typing of IBV is achieved through genetic evolutionary analysis of the S1 subunit sequence. Due to mutations and recombination of the S1 subgene, new IBV genotypes and serotypes emerge; hence, IBV serology and genetic typing are also determined by the S1 gene (13, 14). Studies have shown that the receptor-binding domain (RBD) region of the S1 protein plays a crucial role in the virus’s binding process with chicken respiratory organs and kidneys (15, 16). Thus, the S protein is also one of the determinants of IBV’s tissue tropism. The S1 protein serves as the primary immunogenic protein containing various antigenic epitopes that can stimulate the body’s immune response to produce antibodies, thereby providing immune protection for chickens (17). During virus attachment to host cells, the S2 protein acts as an anchor protein, participating in the fusion process of the host cell membrane and helping the virus enter the host cell membrane to infect the host cell (18). Compared to the S1 protein, the S2 protein is relatively conserved, thus offering relatively stronger cross-protection; a cleavage site on the S2 subunit of the Beaudette strain also affects virus fusion and syncytium formation on Vero cells, indicating that the S2 protein is also related to the virus’s cellular tropism (19).

Research has pinpointed specific regions within the S1 and S2 genes that modulate IBV’s affinity for certain tissues and its pathogenicity, with notable effects observed in the kidneys and respiratory system (20). Notably, modifications to certain sites in the hypervariable region of the S1 protein in the M41 strain have been shown to inhibit tracheal colonization (21). Bouwman et al. delved into the interactions of the RBD of spike proteins from two IBV strains exhibiting divergent nephrotropism, aiming to elucidate the underlying determinants of IBV’s renal tropism. They identified a critical region spanning amino acids 99–159 within the QX-type IBV RBD as essential for renal binding (16). Further comparisons of the RBD regions on the S1 subunit of the M41 strain and the QX-type strain by Bouwman et al. revealed that residues 128KIP130 are crucial for kidney tissue binding (22). Nevertheless, the genetic underpinnings of IBV-induced lesions in the muscles and glandular stomach remain elusive and warrant further exploration.

In this study, we aimed to investigate the pathogenic factors of adeno-myogastritis induced by the IBV H120 commercial vaccine and compare its pathogenicity with the 4/91 strain. We utilized reverse genetics techniques to construct recombinant strains with modified S1 and S2 genes, aiming to identify genetic regions affecting glandular and muscular stomach lesions. Our findings provide valuable insights into the pathogenesis of IBV and offer potential strategies for optimizing vaccine strains by targeting specific regions within the S1 and S2 genes to minimize viral load in the glandular and muscular stomachs, ultimately improving poultry health and productivity.

## MATERIALS AND METHODS

### Virus strains and cells

The H120 and 4/91 attenuated live vaccines were purchased from Guangdong Dahuanong Animal Health Co., Ltd. The IBV H120 strain and the recombinant strains used in this study were both propagated in 9-day-old specific pathogen-free (SPF) chicken embryos.

The BSR-T7/5 cells, a cell line that stably expresses T7 RNA polymerase, were generously provided by Professor Youming Zhang from the Helmholtz Institute of Biotechnology at Shandong University. Chicken kidney (CK) cells were derived from 10-day-old SPF chicken embryos. All these cells were cultured in Dulbecco’s modified Eagle’s medium (DMEM) supplemented with 10% heat-inactivated fetal bovine serum and 1% Antibiotic-Antimycotic solution (containing 10,000 I.U./mL of penicillin and 10,000 µg/mL of streptomycin). The cells were incubated at 37°C with 5% CO_2_.

### IBV vaccine strain pathogenicity test

To initially investigate the pathogenic factors of adeno-myogastritis induced by the H120 commercial vaccine, 120 one-day-old SPF chicks were randomly divided into three groups and housed in separate isolators with *ad libitum* access to food and water. At 1 day old, two strains of commercial vaccines were used to inoculate chicks in two challenge groups via the ocular and nasal routes. Each chick in the experimental groups received a dose of 10^5.5^ EID_50_/0.1 mL, which is 10 times the normal immunization dosage used in production. The control group was inoculated with phosphate buffer saline (PBS) in the same volume. After inoculation, chickens were observed daily for signs and mortality. At 4, 9, 16, 23, and 30 days post-infection (dpi), six chickens from each group were randomly selected and euthanized for necropsy, and the severity of lesions in the glandular and muscular stomachs was scored according to the criteria listed in Tables 1 and 2. Tissue samples from the trachea, lungs, kidneys, duodenum, glandular stomach, and muscular stomach of six chickens per group were collected. Quantitative reverse transcription polymerase chain reaction (qRT-PCR) detection was performed to detect the virus load in different tissues and organs of the challenged groups.

**TABLE 1:** Glandular stomach lesion scoring table

Signs	Score
The mucosal morphology is intact, and the condition is healthy, with clear gastric gland papillae.	0
The mucosa appears thinned, the gastric gland papillae are blurred, and there are vesicular protrusions.	1
Mucosal erosion, with most of the gastric gland papillae disappeared.	2
Mucosal erosion and desquamation, complete disappearance of gastric gland papillae, and bleeding.	3

**TABLE 2:** Muscular stomach lesion scoring table

Signs	Score
The gizzard lining is intact, smooth, and undamaged.	0
The gizzard lining shows localized swelling, appearing “blown” in shape, with a slight state of damage.	1
The gizzard lining is partially damaged and ulcerated, with approximately 50% of the area affected.	2
The gizzard lining is largely damaged and ulcerated, with approximately 75% of the area affected.	3
The entire gizzard lining shows extensive, dense damage and ulceration.	4

### Histopathological analysis

The trachea, lung, kidney, glandular stomach, and muscular stomach of chickens from each group were fixed in 4% paraformaldehyde solution to preserve their structure and prevent any damage or degradation. This solution is commonly used in histological studies to fix tissues and maintain their morphology for further analysis. Once the tissues were fixed, they were carefully sliced into thin sections with a thickness of 5 µm. These sections are thin enough to allow for detailed examination of the tissue structure under a microscope, while still being thick enough to provide sufficient tissue material for analysis. After slicing, the tissue sections were then stained with hematoxylin and eosin (H&E) to enhance their visibility and contrast under a light microscope.

### Plasmid construction and viral rescue

Utilizing the reverse genetics system and techniques established in our laboratory for the H120 strain, we replaced the S, S1, and S2 genes of the H120 vaccine with the corresponding genes from the 4/91 and Beaudette strains. Through these interventions, we successfully constructed and rescued six recombinant strains that are capable of stable transmission and consistent expression of the donor strain’s antigenic genes. These strains include the rH120 strain, rH120-ΔS/Beaudette strain, rH120-ΔS1/Beaudette strain, rH120-ΔS2/Beaudette strain, rH120-ΔS1/491 strain, and the rH120-ΔS2/491 strain. Our approach involved the use of the Red/ET homologous recombination method, as delineated in our prior work (23, 24), facilitated by *Escherichia coli* DH10B cells expressing the Redα/Redβ recombinant protein. The gene sequences of the Beaudette and 4/91 strains were downloaded from the NCBI database. The Beaudette strain (an attenuated lab-adapted strain) has GenBank accession number MZ368698.1, and the 4/91 strain (also known as CR88; a live vaccine strain) has accession number KF377577.1. The S, S1, and S2 genes from both strains were synthesized by Sangon Biotech, flanked by ~40 bp homologous arms. Our recombination strategy consisted of two main steps: initially, “line-loop” recombination was carried out using *E. coli* DH10B gyrA462 that expresses the Redα/Redβ recombinant protein, enabling the substitution or integration of the ccdB-amp screening gene into the expression vector. In the subsequent step, the ccdB-amp screening gene was effectively replaced by either the S1 or S2 gene within the modified *E. coli* DH10B strain, resulting in the isolation of the correct recombinant through stringent ccdB counter selection.

Following the manufacturer’s protocols, the recombinant IBV plasmid was co-transfected with pVAX1-H120-N into BSR-T7/5 cells using Lipofectamine 3000 transfection reagent (Thermo Fisher Scientific, USA). After a 4-hour incubation at 37°C, the supernatant was discarded, and the cells were washed twice with PBS. Fresh DMEM medium, supplemented with 2% FBS and 1% antibiotics, was then added, and the culture was continued for an additional 72 hours. Subsequent to three freeze-thaw cycles, the cell lysates were inoculated into 9-day-old SPF chicken embryos. Forty-eight hours later, the allantoic fluid was harvested from these embryos. After two generations of blind passage in chicken embryos, allantoic fluid was collected. Following the protocol of the Novizan Viral Genomic RNA Extraction Kit, 200 µL aliquots of viral suspension from rH120 strain, rH120-ΔS/Beaudette strain, rH120-ΔS1/Beaudette strain, rH120-ΔS2/Beaudette strain, rH120-ΔS1/491 strain, and rH120-ΔS2/491 strain were subjected to RNA extraction. Primers H120-S-F (AGTGTGGTAAGTTACTGGTAAG) and H120-S-R (GGACGTGGGACTTTGGATCA), designed based on the 3'-end sequence of IBV H120 1ab gene and 5'-end sequence of IBV H120 3a gene, were used to detect full-length S gene expression. The thermal cycling protocol was as follows: 50°C for 30 min (reverse transcription); 94°C for 4 min (initial denaturation); followed by 32 cycles of 94°C for 30 s, 55°C for 45 s, and 72°C for 1 min 20 s; with a final extension at 72°C for 5 min; terminated at 4°C. RT-PCR amplification products were analyzed by 1% agarose gel electrophoresis using a gel documentation system. PCR products were subsequently sequenced.

### Western blot analysis

The recombinant viruses were inoculated into the allantoic cavities of 9- to 11-day-old SPF chicken embryos. Allantoic fluid was harvested 48 hours post-infection and processed for protein analysis. For western blotting, samples were mixed with loading buffer, denatured at 100°C for 8 min, and separated on a 10% SDS-PAGE gel (90 V for 20 min, then 120 V for 40 min). Proteins were transferred to ethanol-activated polyvinylidene fluoride (PVDF) membranes using a semi-dry system (220 mA, 60 min) in a “sandwich” configuration. Membranes were blocked with rapid blocking buffer for 30 min after tris buffered saline tween (TBST) rinsing, followed by overnight incubation at 4°C with anti-IBV N monoclonal antibody (B819M, GeneTex; 1:5,000 dilution). After three TBST washes (10 min each), membranes were incubated with HRP-conjugated goat anti-mouse secondary antibody (1:10,000 in TBST) for 40 min. Signals were detected using the Yeasen ECL kit, with molecular weights validated against a prestained protein ladder via chemiluminescence imaging.

### Recombinant H120 strain pathogenicity test

To preliminarily explore the pathogenic factors of gastritis caused by the H120 commercial vaccine, 315 one-day-old SPF chickens were randomly divided into seven groups (*n* = 45), including a control group and six recombinant H120 strain experimental groups. These chickens were raised in seven isolator units, with free access to food and water.

At the age of 1 day, six experimental groups of chickens were inoculated via eye drop with six strains of recombinant H120 virus. The dose for each inoculation was set at 10^5^ EID_50_/0.2 mL, equivalent to 10 feather portions of the production immune dose. After inoculation, the state of the chickens was observed and recorded daily, with a focus on changes associated with gastritis. Main observations included clinical signs such as depression, tracheal rales, breathing with neck outstretched and mouth open, tearing, etc., as well as mortality. At 4, 9, 16, 23, and 30 dpi, six chickens from each group were randomly selected, sacrificed, and dissected for observation, and the degree of glandular stomach and muscular stomach lesions in each group was scored according to the standards in Tables 1 and 2. Tissue samples from the glandular stomach and muscular stomach were collected, and nucleic acid was extracted from these tissue samples. Virus loads in the glandular stomach and the muscular stomach were detected by qRT-PCR.

### RNA extraction and reverse transcription

Isolate tissue samples and immediately snap-freeze them in liquid nitrogen. Store at −80°C until further use. Thaw the tissue samples on ice and homogenize them using a mechanical homogenizer or a pestle and mortar with ceramic beads. Add TRIzol reagent to the homogenized tissue samples and extract total RNA according to the manufacturer’s instructions. Purify the RNA using an RNeasy mini kit or equivalent, following the manufacturer’s protocol. Elute the RNA in RNase-free water. Reverse transcribes the RNA into cDNA using a reverse transcription kit, following the manufacturer’s protocol. Dilute the cDNA to a suitable concentration for qPCR analysis.

### Viral load determination

The detection of viral copy number in tissues was determined using quantitative PCR following reverse transcription. Viral load quantification in each organ was performed using SYBR Green real-time PCR and standard curves. Specifically, two primers were utilized:

F: 5′-CCTCTAAGGGCTTTTGAG-3′

R: 5′-GTCACTGTCTATTGTATGTC-3′.

These primers amplified a 144 bp PCR product from IBV 5'UTR. The thermal cycling parameters were configured as follows: an initial denaturation at 94°C for 2 min, followed by 40 cycles consisting of a denaturation step at 94°C for 15 seconds and an annealing step at 60°C for 30 seconds. The resulting reaction product was then analyzed on a 1% agarose gel and ligated into a PMD-18-T vector (TaKaRa, Biotechnology, Dalian, China) to transform TOP10 competent cells. Positive clones were sequenced, and plasmids were extracted. The DNA concentration was calculated by measuring the absorbance at 260 nm. Using this DNA concentration, the plasmid copy number was determined with the formula: copies/µL = 6.02 × 10^23^ (copies/mol) × DNA concentration (g/µL)/molecular weight (MW) (g/mol). Serial 10-fold dilutions ranging from 10^3^ to 10^10^ copies of the purified plasmid were prepared in duplicate to generate a standard curve.

### Statistical analysis

Data are expressed as means ± SDs. All data were checked for normality and homogeneity of variance before performing statistical comparisons. Between-group comparisons were conducted using a one-way analysis of variance in GraphPad Prism 9 software. Differences were considered significant at *P* < 0.05.

## RESULT

### Assessing the pathogenicity of the H120 strain

From day 4 post-infection onward, chicks in both challenge groups began displaying signs of lethargy. Specifically, within the H120 group, there was one death on day 9, two deaths on day 10, and an additional death on day 11 post-infection. No mortality was observed in the 4/91 and control group.

**Fig 1:**
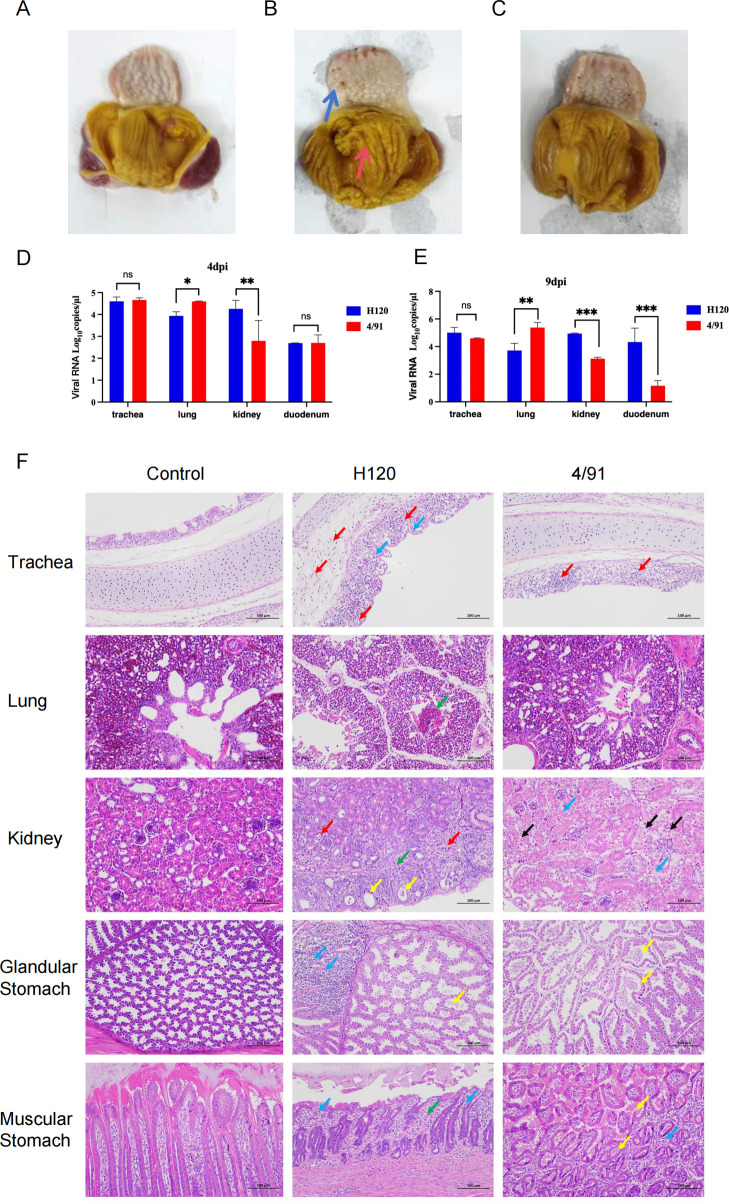
Representative images of severe stomach enlargement and congestion induced by H120 to SPF chickens. One hundred twenty 1-day-old SPF chickens were infected with H120 and 4/91(10^5.5^ EID_50_/0.1 mL per chicken) or PBS via the nose and eye. The stomach was macroscopically examined at 7 days post-challenge. Representative images from the uninfected group, H120-infected SPF chickens, and 4/91-infected SPF chickens are shown in panels A, B, and C, respectively. (**A**) Negative control (PBS-inoculated). (**B**) H120-infected chickens showing marked enlargement, congestion, and mucosal mottling. *Blue arrows*: indistinct gastric gland papillae and vesicular protrusions in proventriculus; *red arrows*: extensive muscular stomach ulceration. (**C**) 4/91-infected group. (**D and E**) Viral RNA copies in tissues at 4 dpi (**D**) and 9 dpi (**E**). (**F**) Histopathology (200×): Trachea: *red arrow*—scattered lymphocyte infiltration; *blue arrow*—mucosal epithelial hydropic degeneration (cytoplasmic pallor/loosening). Lung: *green arrow*—moderate parabronchial hemorrhage. Kidney: *yellow arrow*—mild cortical tubular dilation with intraluminal eosinophilic flocculent material; *green arrow*—mild interstitial connective tissue hyperplasia; *red arrow*—focal lymphocyte infiltration; *black arrow*—tubular epithelial necrosis (nuclear pyknosis/hyperchromasia). Glandular stomach: *yellow arrow*—eosinophilic flocculent material in glandular lumina; *blue arrow*—lamina propria lymphoid aggregates. Muscular stomach: *green arrow*—mild stromal connective tissue hyperplasia; *blue arrow*—scattered lymphocytes; *yellow arrow*—keratin-like material in glands/interstitium. Bars represent mean ± SD; *n* = 6 chicks per group.*P ≤ 0.05, **P ≤ 0.01, and ***P ≤ 0.001 (one-way ANOVA with Tukey’s post hoc comparisons).

The final results revealed that, in the H120 group, eight chicks had lesions in the muscular stomach, and seven chicks exhibited lesions in the glandular stomach. In comparison, the 4/91 group had six chicks with muscular stomach lesions and five with glandular stomach lesions (Table 3). Pathological changes in the muscular stomach included partial damage and ulceration of the gizzard lining, while the glandular stomach showed a thinned mucosa, indistinct gastric gland papillae, and vesicular protrusions in both infected groups (Fig. 1A through C).

**TABLE 3:** Necropsy results of the pathogenicity test of IBV commercial vaccine strain in 1-day-old SPF chickens

Group	IBV strain	Number	Glandular gastropathy	Muscular gastropathy	Deaths
H120	H120	10	9	11	4
4/91	4/91	10	5	6	0
Control	Physiological saline	10	0	0	0

To compare the pathogenicity of vaccinal strains, we measured viral loads in the trachea, lungs, kidneys, and duodenum at 4 and 9 dpi. The results revealed that in the kidneys, the H120 group exhibited significantly higher viral loads compared to the 4/91 group at 4 dpi and 9 dpi. Conversely, in the lungs, the 4/91 group showed significantly higher viral loads than the H120 group. Additionally, the IBV H120 strain demonstrated higher viral loads in the duodenum at 9 dpi. In the trachea, the two viruses had similar viral loads at 4 dpi and 9 dpi (Fig. 1D and E). Histopathological analysis of H&E-stained sections revealed systemic histopathological lesions across multiple organs in both vaccine groups administered a 10-fold overdose. Notably, the H120-vaccinated group exhibited focal lymphocytic aggregates within the glandular stomach (blue arrows, Fig. 1F), indicative of localized immune infiltration. The lamina propria of the muscular stomach exhibited focal areas with irregularly arranged gastric glands, accompanied by mild connective tissue hyperplasia in the stroma (green arrows). These findings indicate that the two viruses have similar pathogenicity but different tissue tropisms.

### The H120 strain causes persistent stomach inflammation

**Fig 2:**
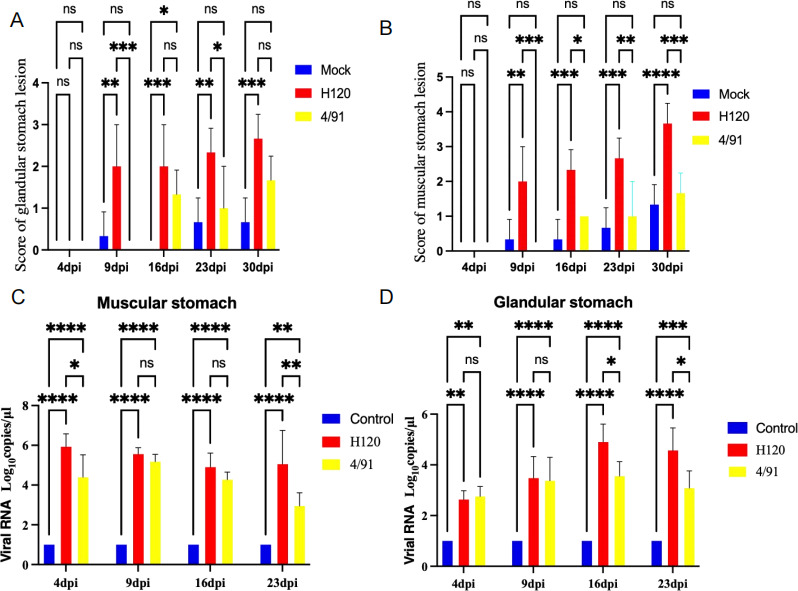
The details of the assessment of lesions in the glandular and muscular stomachs of chickens from two challenged groups and a control group at various days post-challenge with H120 or 4/91. (A) The figure presents an evaluation of glandular stomach lesions in chickens from two challenged groups and a control group at 4, 9, 16, 23, and 30 days post-challenge with H120 or 4/91. (B) The evaluation of muscular stomach lesions in chickens from the same challenged groups and control group at 4, 9, 16, 23, and 30 days post-challenge with H120 or 4/91 is also depicted. (C) The graph shows viral RNA copies of the glandular stomach at 4, 9, 16, and 23 days post-challenge with H120 or 4/91 in different groups. (D) Viral RNA copies of the muscular stomach at 4, 9, 16, and 23 days post-challenge with H120 or 4/91 in different groups are also represented. Bars represent mean ± SD; *n* = 6 chicks per group.*P ≤ 0.05, **P ≤ 0.01, ***P ≤ 0.001, and ****P ≤ 0.0001(one-way ANOVA with Tukey’s post hoc comparisons).

To further investigate the pathogenic effects of the H120 strain on chicken stomachs, symptom scores were collected at 4 dpi, 9 dpi, 16 dpi, 23 dpi, and 30 dpi. Initially, at 4 dpi, there was no significant difference in the glandular stomach lesion scores between the two challenged groups and the control group. However, by 9 dpi, the H120 group exhibited a significant increase in lesion scores (*P* < 0.001) compared to the control group and also displayed a significant difference (*P* < 0.05) when compared to the 4/91 group. At 16 dpi, 23 dpi, and 30 dpi, the H120 group maintained the highest scores, demonstrating a persistent significant difference from the control group. Meanwhile, the 4/91 group only achieved a significant difference (*P* < 0.05) compared to the control group at 16 dpi (Fig. 2A). Similarly, the muscular stomach lesions followed the same trend as the glandular stomach lesion scores. At 9 dpi, 16 dpi, 23 dpi, and 30 dpi, the H120 group consistently had the highest scores, showing a significant difference from the control group. Meanwhile, the 4/91 group displayed an increasing trend in scores but did not show a significant difference compared to the control group at 4 dpi, 9 dpi, 16 dpi, 23 dpi, and 30 dpi (Fig. 2B).

In order to understand the pathogenicity of the H120 strain on chicken stomachs, we measured the viral loads in both the glandular and muscular stomachs at different time points. In the glandular stomach, at 16 dpi and 23 dpi, the viral load of the H120 group was significantly higher than that of the 4/91 group (Fig. 2C). Concurrently, in the muscular stomach, the viral loads of the H120 group were significantly higher than those of the 4/91 group at 4 dpi and 23 dpi (Fig. 2D). This high viral replication of H120 in the stomach could be the underlying reason for its pathogenicity in chickens.

### The replacement of S1 reduces H120 pathogenicity in the chicken’s muscular stomach

**Fig 3:**
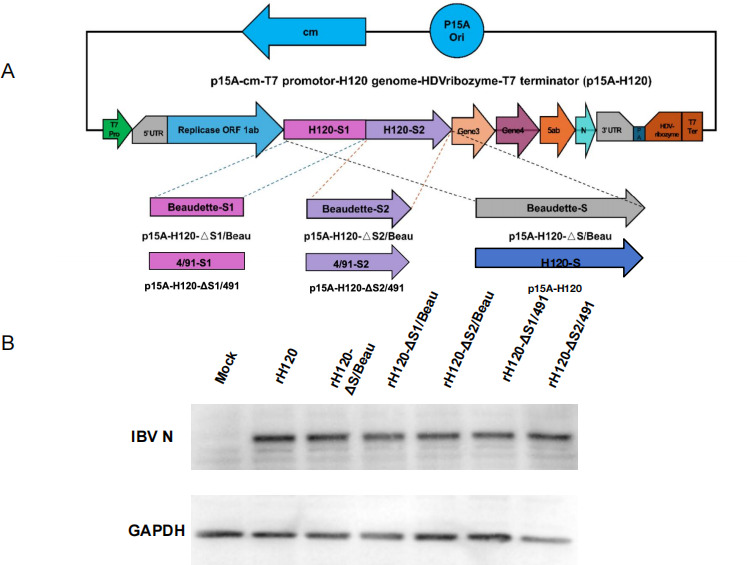
Utilizing the reverse genetics system specific to the H120 strain, we substituted the S, S1, and S2 genes with their counterparts from the 4/91 and Beaudette strains. (A) This meticulous procedure yielded six distinct recombinant strains: rH120, H120-ΔS/Beaudette, H120-ΔS1/Beaudette, H120-ΔS2/Beaudette, H120-ΔS1/491, and H120-ΔS2/491. Notably, all these recombinant strains maintain stable transmissibility and consistently express the antigen genes derived from the donor strains. (B) To detect the N protein of IBV in chicken embryos, we employed western blot analysis, using GAPDH as a reference for standardization.

Current studies indicate that the genes responsible for the tissue and cellular tropism of IBV are primarily found on the S gene. In previous experiments, strain 4/91 caused less severe lesions in the glandular stomach, and the Beaudette strain shares high homology with the H120 strain but is not pathogenic. Therefore, strains 4/91 and Beaudette were chosen as donors for the recombinant viruses. Using our laboratory’s reverse genetics system and techniques, different fragments of the donor S gene were replaced to identify the genetic regions affecting glandular stomach lesions. Five recombinant virus strains were constructed and rescued, named rH120, rH120-ΔS/Beaudette strain, rH120-ΔS1/Beaudette strain, rH120-ΔS2/Beaudette strain, rH120-ΔS1/491 strain, and rH120-ΔS2/491 strain (Fig. 3A). The CK cells were infected with the recombinant viruses, and western blot analysis was used to detect IBV N protein, confirming the successful generation of all six recombinant viruses (Fig. 3B).

To determine the pathogenicity of all six recombinant viruses, 210 one-day-old SPF chickens were randomly allocated into seven groups: a control group and six groups for the recombinant H120 strains. Each of the six recombinant H120 strain groups was inoculated with the respective recombinant H120 strain via the nasal and ocular routes. The control group received PBS as a substitute. Mortality in chickens began at 3 dpi for the rH120 strain, rH120-ΔS/Beaudette strain, and rH120-ΔS2/Beaudette strain. The rH120-ΔS1/Beaudette and rH120-ΔS1/491 strains observed mortality starting at 5 dpi, while no deaths occurred in the rH120-ΔS1/Beaudette strain (Table 4).

**TABLE 4:** Necropsy results of the recombinant strains in 1-day-old SPF chickens

Group	IBV strain	Number	Glandular gastropathy	Muscular gastropathy	Deaths
rH120	rH120	15	6	10	4
H120-ΔS/Beau	H120-ΔS/Beaudette	15	6	7	1
H120-ΔS1/Beau	H120-ΔS1/Beaudette	15	4	7	0
H120-ΔS2/Beau	H120-ΔS2/Beaudette	15	5	6	1
H120-ΔS1/491	H120-ΔS1/491	15	3	5	0
H120-ΔS2/491	H120-ΔS2/491	15	5	7	1
Control	Physiological saline	15	0	0	0

**Fig 4:**
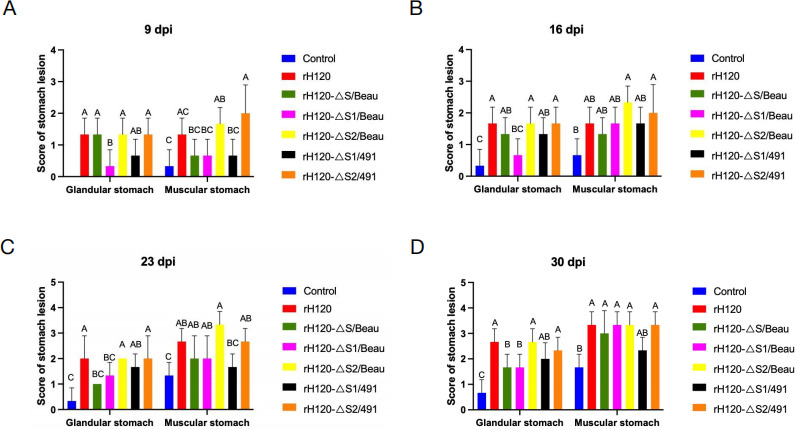
Details the assessment of lesions in the glandular and muscular stomachs of chickens from six challenged groups and a control group at various dpi. Lesion scores at (A) 9, (B) 16, (C) 23, and (D) 30 dpi are presented. Bars represent mean ± SD; *n* = 6 chicks per group. Two groups marked with different letters indicate a significant difference (*P* < 0.05; one-way ANOVA with Tukey’s post hoc comparisons).

To more comprehensively assess the pathogenic impact of the recombinant viruses on chicken stomachs, symptomatic scores were meticulously documented at intervals of 4 dpi, 9 dpi, 16 dpi, 23 dpi, and 30 dpi. No gastric lesions were observed in any experimental group at 4 dpi, with lesion scoring indices remaining at 0 across all cohorts. Notably, the glandular stomach lesion scores for the rH120-ΔS1/Beaudette and rH120-ΔS1/491 variants were consistently lower than those observed in the H120 strain at 9 dpi, 16 dpi, 23 dpi, and 30 dpi (Fig. 4A through D). Similarly, the rH120-ΔS/Beaudette variant exhibited reduced glandular stomach lesion scores relative to rH120, at 23 dpi and 30 dpi (Fig. 4C and D). In contrast, the glandular stomach lesion scores for the rH120-ΔS2/Beaudette and rH120-ΔS2/491 groups remained statistically indistinguishable from those of the rH120 group throughout the monitoring period, from 9 dpi, 16 dpi, 23 dpi, and 30 dpi. The scores for muscular stomach lesions across the six challenge groups were complex and did not reveal a uniform pattern (Fig. 4A through D). Collectively, these findings suggest that the substitution of the S1 gene has the potential to mitigate the pathogenicity of the H120 strain on the glandular stomach.

### The H120 S1 gene influences viral replication in the chicken stomach

**Fig 5:**
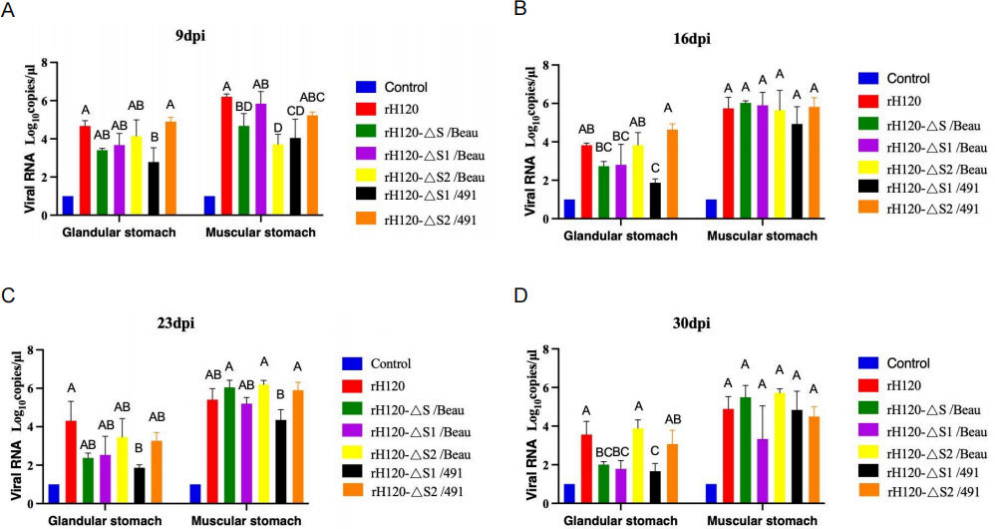
IBV genome loads in glandular and muscular stomach collected at 9, 16, 23, and 30 dpi. (**A**) The mean viral RNA copies of the glandular and muscular stomach at 4 dpi in different groups. (**B**) The mean viral RNA copies of the glandular and muscular stomach at 9 dpi in different groups. (**C**) The mean viral RNA copies of the glandular and muscular stomach at 16 dpi in different groups. (**D**) The mean viral RNA copies of the glandular and muscular stomach at 23 dpi in different groups. Bars represent mean ± SD; *n* = 6 chicks per group. Two groups marked with different letters indicate a significant difference (*P* < 0.05; one-way ANOVA with Tukey’s post hoc comparisons).

To investigate the variations in tissue viral loads among chickens infected with six different strains, samples from the glandular and muscular stomachs were randomly collected from three chickens at 9 dpi, 16 dpi, and 30 dpi to quantify the IBV viral load in these tissues. As illustrated in Fig. 5A, at 9 dpi, the viral loads of the rH120-ΔS1/491 group were significantly lower than those of the rH120 group in both the glandular and muscular stomachs. In contrast, the viral loads of the rH120-ΔS/Beau, rH120-ΔS2/Beau, and rH120-ΔS1/491 groups were significantly lower in the muscular stomach compared to the rH120 group. The viral loads of the rH120-ΔS1/Beau and rH120-ΔS2/491 groups did not show a significant change compared to the rH120 group. At 16 dpi, the viral loads of the rH120-ΔS1/491 group were significantly lower than those of the rH120 group for the glandular stomach. However, for the muscular stomach when compared to the H120 group, there was no significant difference in the viral loads among the other four recombinant virus groups (Fig. 5B). By 23 dpi and 30 dpi, the viral loads of the rH120-ΔS/Beau, rH120-ΔS1/Beau, and rH120-ΔS2/491 group were significantly lower than those of the rH120 group in the glandular stomach (Fig. 5C and D). Meanwhile, the viral loads of the rH120-ΔS/Beau group were significantly lower than those of the rH120 group in the muscular stomach at 14 dpi (Fig. 5D). The results indicate that replacement of S1 can affect viral replication in the chicken glandular stomach.

## DISCUSSION

This study aims to investigate the primary pathogenic factors of glandular and muscular stomach inflammation induced by the IBV H120 strain in poultry production. Compared to the 4/91 strain, the H120 strain exhibits a stronger tropism for both the chicken’s glandular and muscular stomachs. Replacing the S1 gene of the H120 strain resulted in decreased pathogenicity in the chicken’s muscular stomach, indicating that the S1 gene of the H120 strain appears to have a significant influence on viral replication within the chicken’s stomach and the subsequent development of lesions in both the muscular and glandular regions.

In flocks vaccinated with the H120 vaccine, an increased incidence of glandular and muscular stomach inflammation in chickens was observed. Given that farming conditions and other environmental factors were kept constant, and the possibility of other pathogen infections was ruled out, this phenomenon is likely related to the use of the vaccine. Pathogenicity testing using the H120 vaccine reproduced the signs of glandular and muscular stomach inflammation in chickens, with the 4/91 vaccine used as a control and a challenge dose of 10^5.5^ EID_50_/0.1 mL. The results showed that the H120 strain tended to cause more respiratory system damage, while the 4/91 strain led to kidney damage. Further observations revealed that the H120 strain caused more significant lesions in the chicken’s glandular and muscular stomachs, consistent with observations from production sites.

RNA viral replication (for example, avian influenza virus) in tissues (lung) is closely related to its tissue tropism (25, 26). Tissue tropism refers to the preference of a virus to infect and replicate in specific host cells or tissue types (27, 28). Viruses rely on the biosynthetic mechanisms of host cells for replication, involving processes such as viral entry into host cells, release of genetic material, and utilization of the host cell’s synthetic machinery to produce new viral particles, ultimately leading to the destruction or death of the host cell. Tissue tropism is influenced by various factors, including viral surface proteins, receptors on the host cell surface, and intracellular signaling pathways within the host cell. For example, some viruses may preferentially infect and replicate in cells with specific receptors, which are differently expressed in various tissue types, thereby determining the virus’s tissue tropism. In our experiments, compared to the 4/91 strain, the H120 strain demonstrated stronger viral replication capability in the chicken’s glandular and muscular stomachs and also caused more severe glandular and muscular stomach lesions. This discovery provides important clues for further research into the pathogenic mechanism of IBV and optimization of vaccine strains.

In this study, the S1-recombinant virus demonstrated attenuated pathogenicity in inducing glandular stomach inflammation in chickens. The S protein, a key structural component of coronaviruses, plays a crucial role through its S1 subunit’s RBD, enabling the virus to recognize and infect host cells (29). In the case of IBV, a unique coronavirus, the highly variable regions of the S protein, especially the RBD, are closely linked to the virus’s pathogenicity, tissue tropism, and serological diversity (30). The amino acid sequence variations in the RBD region directly affect the virus’s ability to bind to host cell receptors. Differences in the RBD regions among various IBV strains may lead to different binding affinities to specific receptors, determining the virus’s affinity for different cells and tissues, and thereby affecting the diversity of clinical manifestations it causes, such as respiratory infections, kidney infections, or reproductive system infections (22). For example, the M41 strain’s RBD tends to bind to α2,3-sialic acid receptors (16), while the QX strain’s RBD region, specifically amino acids like KIP at positions 110-112, is crucial for kidney infection (16). These variations not only determine the virus’s tissue tropism, but they could also be key factors in its pathogenicity.

In this study, considering that the 4/91 strain causes milder lesions in the glandular and muscular stomachs, and the Beaudette strain exhibits lower pathogenicity and high homology with the H120 strain, these two strains were selected as donors for constructing recombinant strains. Using reverse genetics technology, we replaced different fragments of the donor S gene to determine the genetic regions affecting lesions in the glandular and muscular stomachs, constructing six recombinant strains. Pathogenicity assessment experiment of the recombinant viruses revealed that compared to the H120 strain, the ΔS/Beaudette strain, ΔS1/Beaudette strain, and ΔS1/491 strain had reduced chicken glandular stomach lesions. This highlights the central role of the S1 gene in the pathogenic process of IBV in chicken glandular stomachs and suggests that mutations may lead to decreased viral pathogenicity. For muscular stomach lesion scores, the recombinant viruses did not show differences, implying that the S gene may not be directly related to muscular stomach pathogenicity. Viral load analysis showed that at 4 dpi, the ΔS/Beaudette strain, ΔS1/Beaudette strain, and ΔS1/491 strain had significantly lower viral loads in the glandular stomach compared to other strains. Comprehensive scoring of glandular and muscular stomachs and viral load results indicated that the S1 gene had the most significant effect in reducing glandular stomach lesions. We speculate that the genes affecting glandular stomach lesions are primarily located on the S1 gene, providing important clues for subsequent optimization studies of the H120 vaccine. Notably, the rH120-ΔS2/Beau cohort exhibited significantly reduced viral loads (*P* < 0.05) in the muscular stomach compared to the rH120 control group at 9 days post-inoculation, suggesting potential regulatory effects of the S2 glycoprotein on early-stage viral replication within this gastrointestinal compartment.

It is important to acknowledge that a challenge dose 10-fold higher than the standard vaccination dose was used to accelerate observable pathology. While this approach facilitated the identification of S1-mediated tropism, it may amplify pathogenic phenotypes beyond those seen in field conditions. Critically, this finding underscores a vital practical implication: poultry producers must strictly adhere to recommended vaccine dosages. Overdosing with H120 vaccines, as mimicked in this study, may exacerbate glandular stomach inflammation due to the inherent tropism of the H120. Future studies using standard doses are warranted. Additionally, while qRT-PCR robustly indicates replication differences, immunohistochemical detection of viral proteins would strengthen evidence for tissue-specific replication. These limitations highlight the need for cautious interpretation of the experimental pathogenesis model.

The results of this study have profound implications for the poultry industry, offering important insights into the pathogenesis of IBV and potential strategies for optimizing vaccine strains. Targeting specific regions within the S1 gene could reduce viral loads in glandular and muscular stomachs, thereby enhancing poultry health and production capacity. These findings contribute to a deeper understanding of the genetic basis of IBV-induced lesions and offer new avenues for developing improved vaccine strains. This study emphasizes the necessity of continuing to explore the genetic factors of IBV pathogenicity, with future research focusing on identifying other genetic determinants of IBV tropism and pathogenicity, as well as exploring targeted interventions to improve the safety and effectiveness of IBV vaccines. Additionally, understanding the interaction between IBV and host immune responses may lead to novel antiviral strategies and treatments for IBV infection.

In summary, this study highlights the critical role of the S1 gene in the pathogenicity of the IBV H120 strain, particularly in causing lesions in chicken glandular stomachs. The insights gained from this study could pave the way for developing improved IBV vaccines, reducing the incidence of adverse reactions associated with vaccination, and enhancing poultry health and production efficiency. Ongoing research in this area is crucial for refining our understanding of IBV pathogenicity and devising effective strategies to mitigate its impact on the poultry industry.
